# Effect of dust accumulation on the performance of photovoltaic modules for different climate regions

**DOI:** 10.1016/j.heliyon.2023.e23069

**Published:** 2023-11-30

**Authors:** Mahnoor Rashid, Muhammad Yousif, Zeeshan Rashid, Aoun Muhammad, Mishal Altaf, Adil Mustafa

**Affiliations:** a U.S. Pakistan Centre for Advanced Studies in Energy (USPCAS-E), National University of Sciences and Technology (NUST), Islamabad, Pakistan; b Department of Electrical Engineering, The Islamia University of Bahawalpur, Pakistan; c Department of life Sciences, University of Warwick, Coventry, United Kingdom

**Keywords:** PV module, Efficiency, Dust accumulation, Cleaning technologies

## Abstract

In the past decade, solar photovoltaic (PV) modules have emerged as promising energy sources worldwide. The only limitation associated with PV modules is the efficiency with which they can generate electricity. The dust is the prime ingredient whose accumulation on the surface of PV impacts negatively over its efficiency at a greater rate. This research aims to explore the effects of dust accumulation on the energy output and operating temperature of polycrystalline silicon PV panels situated in two different climatic regions of Pakistan, *i.e.*, Islamabad and Bahawalpur. In both the regions, one PV module is kept in ambient environment for six weeks to allow dust to deposit over its surface and perform experimental analysis with one clean module as reference for performance comparison. After six weeks of atmospheric exposure, dusty modules displayed significantly smaller efficiency as a function of different dust densities in the two regions. Dust samples from both cities are collected and analyzed to evaluate their structural attributes and composition. The PV module in Islamabad region had a dust layer over its surface with a density of 6.388 g/m^2^ and its efficiency was reduced by 15.08%. In Bahawalpur region, the dust density was observed to be 10.254 g/m^2^ which caused the output power to be slashed by 25.42%. Temperature analysis of modules shows that dust increases their temperatures which is also a quantity responsible for lower PV power generation with same amount of irradiance. The research findings are crucial for determining and predicting PV power degradation in two different atmospheres and determining the schedule of cleaning cycle.

## 1 Introduction

Renewable energy sources are becoming a popular option for meeting the rise in the energy demands [1]. Governments around the globe are urging policy reforms to encourage renewable energy deployment [2], [3], [4]. Thus, by 2022, the amount of renewable energy installed worldwide is more than double than that of 2011 [5]. Solar energy is the most viable option among renewable energy sources. It is also relatively inexpensive, easy to install and readily accessible [6], [7]. Approximately, $1.8\times 10^{11}$ MW of energy is transmitted to the earth's surface every second through solar radiation [8]. This amount represents only a fraction of world's energy consumption [9].

The PV technology is getting expansion at a higher pace due to which its investigation for the efficiency affecting factors is becoming more demanding. PV modules are influenced by internal and external factors like structural properties, aging, irradiance, partial shading, smoke, module temperature and most importantly dust deposition. As a consequence of deposition of dust layer carried by the wind, PV modules are adversely affected in their ability to conduct solar radiation. Moreover, absorbing and scattering sunlight directly alters the earth's temperature by reducing the intensity of sunlight [10]. The loss in energy yield due to dust deposition depends on the parameters of the locality such as atmosphere, topography *e.g.* plain or hilly, personnel behavior and type of PV module. PV modules should be cleaned periodically to minimize these losses.

The research on PV modules in the context of dust effect on their efficiency has been going on actively over the past decade. Most of the research is conducted in the areas which are less rainy and surrounded by deserts due to which sand content in air is high and dust deposition is fast. Furthermore, almost zero rain frequency in these areas prohibits natural cleaning of the module's surface and therefore, a periodic cleaning setup and schedule is essential to minimize inevitable power loss. Sarver et al. concluded that even after only two months of outdoor exposure without cleaning, energy yield can be reduced by 6.5%. Furthermore, results from SEM analysis and XRD revealed that sand particle size and morphology may vary by region [11]. There is a high dust accumulation on PV panel surfaces in desert areas [12], [13]. Abbas et al. reported that a dust storm can reduce PV module power output by 20%, and long-term exposure can reduce it by 50%. Analyzing the impact of dust in this climate is challenging compared to others [14]. Based on the SEM analysis, Adinoyi et al. reported that the dust particles have irregular shapes and sizes. Dust particles of various sizes and shapes have an influence on shading on PV module surfaces, thereby affecting the power output of PV modules [15]. Rajput et al. conducted an experimental study [16] to investigate the effect of dust particles deposited on PV modules. They examined periodic personnel activities, PV sizing, design protocols and irradiance levels and concluded that dust significantly reduces solar PV modules' efficiency. Sulaiman et al. concluded that PV performance could be reduced by over 85% by dirt accumulation [17]. Kaldellis et al. conducted a study in Athens which is an environmentally offensive region and found that dust alters the PV performance differently depending on the site [18]. Kazem et al.'s experimental research based on a concept about dust deposition's effect on PV module's performance showed a reduced output power by almost 40%. XRD results showed that Quartz silicates and calcium oxides were main components of dust and SEM analysis showed that smaller dust particles form large clusters [19]. A numerical investigation on the effects of dust deposition on ground-mounted PV panels by Lu et al. concluded that the dust deposition rates are higher when the panels are horizontal over the ground [20]. Jaszczur et al. investigated that the deposition of dust potentially increases the temperature of the PV module and lowers the output energy yield [21]. It is useful to analyze PV system production losses by determining the dust deposition rate and frequency of rain. Weber et al. analyzed the annual loss in energy from dust accumulation on PV modules and processed climatic data to obtain information about rainfall frequency, in Maxico city [22]. The results showed that up to 15% of PV system output might be lost due to dust accumulation during rainless 60 days. In Egypt, Hassan et al. analyzed two PV technologies and compared their efficiency reductions [23]. There was a decrease of 33.5% in the efficiency of polycrystalline silicon PV modules and a decrease of 65.8% in the efficiency of amorphous silicon PV modules. A 70-day experimental analysis by Gholami et al. showed a reduction of 21.47% in the output power of the PV module [24]. Jamali et al. determined the impact of environmental and climate factors on PV modules [25]. The results demonstrated that PV system's efficiency was primarily affected by dust. Charabi et al. applied multiple spatial geographic information system parameters together with fuzzy logic on PV plants and investigated the cumulative effect on the PV performance. According to Gostein et al., dust and other environmental contaminants on PV panels result in loss of potential power [26].

Recent studies report on the errors related to the soiling estimation models. Javier et al. characterized soiling losses by comparing six soiling models on the basis of machine learning and physical approach [27]. Results showed that physical models performed better compared to machine learning models. Several different soiling models have been tested by Shubham et al. at three different study locations to analyze soiling phenomenon [28]. Empirical models showed highest percentage of errors followed by PM-deposition based models while transmittance loss models showed least errors. Isaacs et al. proposed and implemented a methodology using the Coello framework to develop soiling estimation model [29]. In comparison with Coello's unadjusted approach, this model decreased error by approximately 50% when estimating soiling-related power losses. Khalid et al. presented a systematic analysis of dust deposition, impact and mathematical model and proposed sustainable cleaning methods for PV systems in dusty environment. In addition, they discussed how these cleaning mechanisms might face future challenges. [30]

According to the previous literature review and discussions, dust adversely affects PV modules and varies from region to region. Therefore, the dust efficiency loss for specific dust types and models cannot be accurately predicted by a specific practical or theoretical model. Most of the reported research focuses on the effect of a few parameters and the comprehensive chemical analysis of the dust from different regions is also limited. In addition, not much research has been done on how dust deposition affects the module's temperature which is a significant prospect since the efficiency of PV module is reduced with the increase in temperature. Across the globe, dust varies significantly in structure and composition due to topographic, geological and environmental factors so an investigation of PV module's performance for different dust elements is required. In this paper, amount of dust density accumulated on the surface of polycrystalline PV modules and its impact on the electrical performance and operating temperature of the PV modules in two different cities in Pakistan are examined. Samples of dust from both cities are also tested to obtain composition and particle size to be a reference for further assessment of cleaning methods and routines accordingly in both cities. The impact on the operating temperature of the PV module due to dust deposition is observed by the temperature difference between the dirty and clean modules. PV modules are tested at 25 ^∘^C and 60% loading relative to the maximum power point and it has been estimated that, for an increase of every Celsius beyond 25 ^∘^C, the efficiency is reduced by 0.38%. A six-week dust collection experiment is conducted at two sites in Pakistan (Islamabad and Bahawalpur). Dust samples are taken from both sites, and their elements are examined and compared to determine their impact.

This paper is organized as follows. Section 2 discusses the climatology of the test regions. In section 3, the methodology of the research using various measurements and analysis tools is presented. Results on the performance of PV modules and effect of dust are presented and compared in section 4. Finally, the conclusion of the research is drawn in section 5.

## 2 Climatology of test regions

**Figure 1:**
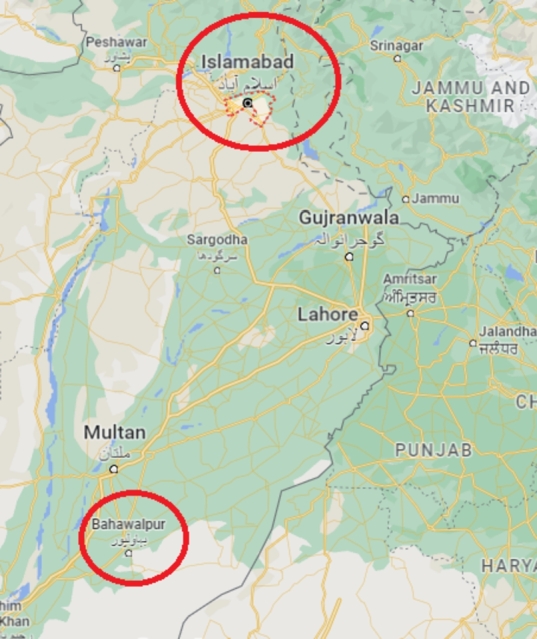
Map of test locations.

One of the locations where the setup is installed lies at 33.6425^∘^ north latitude and 72.9930^∘^ east longitude in Islamabad. Islamabad's climate is clean, pleasant and warm with an average annual temperature of 20.3 ^∘^C (Celsius). The warmest month is June, having an average minimum temperature of 24 ^∘^C and an average maximum temperature of 38 ^∘^C. January is the coldest month in Islamabad having an average minimum temperature of 4 ^∘^C and an average maximum temperature of 18 ^∘^C. Even the driest month contains much rainfall which contributes to cleaning of PV modules. The output of a PV module depends on solar radiation *i.e.*, the more daily hours of sunshine, the more the electric output of the module. Around 3691.11 hours of sunshine are counted in Islamabad annually [31]. The second testing region where the setup is installed lies at 29.33544^∘^ north latitude and 71.6911^∘^ east longitude in Bahawalpur as shown in Fig. 1. Bahawalpur is located in a desert region with a dry climate with virtually no rainfall but with frequent wind and dust storms with an average annual temperature of 26.1 ^∘^C.

The warmest month is June having an average minimum temperature of 29 ^∘^C and an average maximum temperature of 41 ^∘^C. January is the coldest month in Bahawalpur, having an average minimum temperature of 7 ^∘^C and an average maximum temperature of 20 ^∘^C. The daily sunshine hours are significant here because this is a desert region. Hence, this region is much more suitable for solar plant integration in terms of daily hours of sunshine. Bahawalpur has an average of 3852.73 sunshine hours annually [31]. A 100 MW PV power station at Quaid-e-Azam solar Park was established in 2014. Various site-specific climatological and anthropological variables must be evaluated to minimize dust-related energy losses including topography, weather, and human activity. It is not possible to control the climatological parameters however, it is possible to minimize them.

## 3 Methodology

The research progresses by setting up experimental setups at both sites (cities). This experimental setup is designed to analyze the impact of dust in the atmosphere collected from solar panels in both locations having different topography and climatology on the PV module's electrical performance and temperature. Dust samples from both cities are collected and tested to determine the composition of elements and examine particle size to analyze further and compare the impact on the output of PV modules. An outdoor evaluation is done simultaneously at the rooftop of a building in Islamabad and the rooftop of a house in Bahawalpur for six weeks from 1st June to 15th July 2022. The experimental setup consists of a system for PV, dust density measurement, dust composition and chemical analysis. To avoid other weather variables' interference, measurements are taken on clear days under similar conditions. Therefore, the output will clearly show the effects of dust deposition on the performance of PV modules.

### 3.1 Outdoor PV test setup and procedure

**Figure 2:**
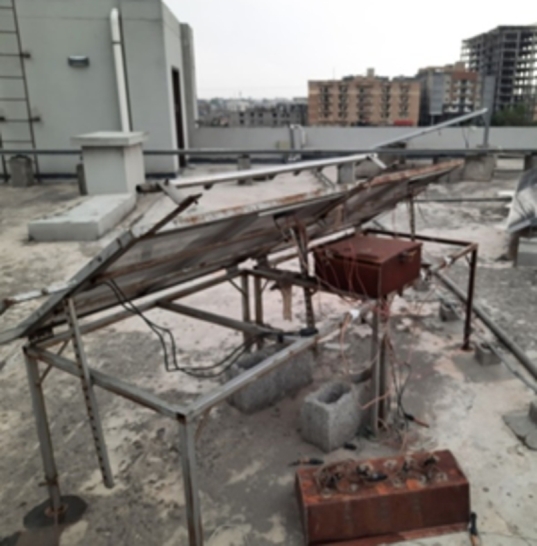
PV module setup at Islamabad.

**Figure 3:**
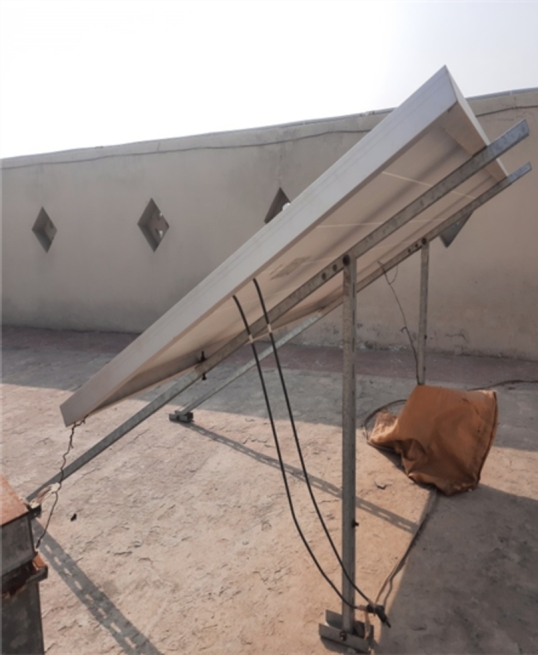
PV module setup at Bahawalpur.

The assembly in the outdoor atmosphere at two sites consists of two polycrystalline PV modules mounted on a south-facing metal stand with a fixed tilt angle of 34^∘^. The power losses of the PV modules are assessed by placing both modules next to each other as shown in Fig. 2 and Fig. 3.

**Figure 4:**
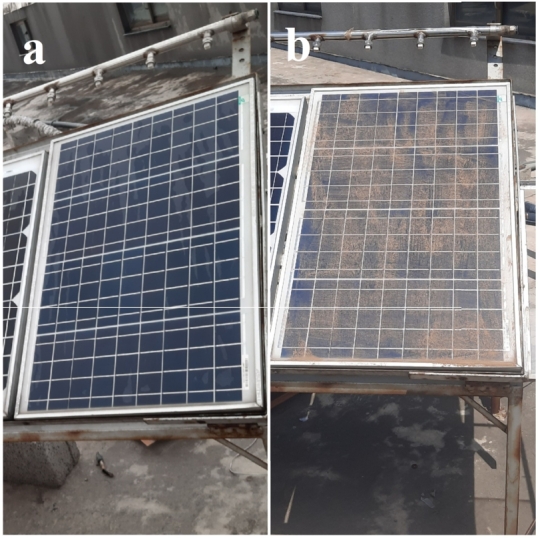
(a) Clean module (b) dirty module at Islamabad.

**Figure 5:**
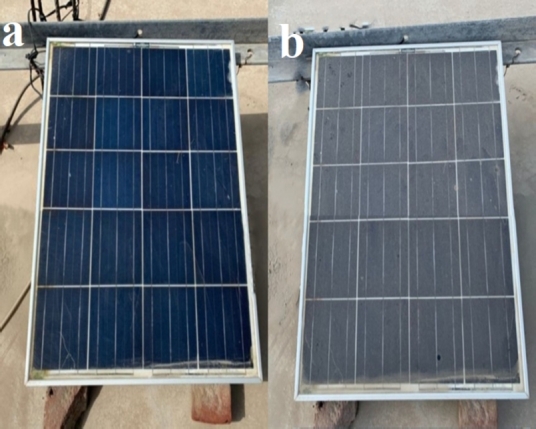
(a) Clean module (b) dirty module at Bahawalpur.

One module of each type is regularly wiped while the other one is allowed to naturally accumulate the dust. The clean and dirty modules at the beginning and end of experiments are shown in Figs. 4(a) and 4(b) in Islamabad region and in Figs. 5(a) and 5(b) in Bahawalpur region respectively. Both PV modules are from Shivgreen Solar System with a manufacturer rating of 40 W and other nominal values as shown in Table 1.

**Table 1:** PV modules' specifications.

Parameters	Specifications
Maximum power (*P*_*max*_)	40 W
Voltage at maximum power (*V*_*mp*_)	17.28 V
Current at maximum power (*I*_*mp*_)	2.31 A
Open circuit voltage (*V*_*oc*_)	21.60 V
Short circuit current (*I*_*sc*_)	2.47 A
Operating temperature	-40 ^∘^C to 90 ^∘^C

The pyranometer is oriented at an angle to the PV modules to measure global solar radiation. Data of maximum current ($I_{max}$) and maximum voltage ($V_{max}$) of both clean and dirty modules is collected three times per week (on clear days). The voltage and current of each module are measured using two digital multimeters (Fluke 317, accuracy: 1.5% for AC and 1% for DC volts). The temperature is recorded using a digital IR thermometer. After collecting all the data of power, efficiencies of PV modules are calculated using the following relationships.

#### 3.1.1 Maximum power

Maximum power is calculated through maximum voltage and maximum current using Eq. (1).

(1) $$P_{max}=I_{max}\times V_{max}$$

Where $P_{max}$ = Maximum Power of the module,

$I_{max}$ = Maximum current of the module,

$V_{max}$ = Maximum voltage of the module.

#### 3.1.2 Percentage reduction in output power

The percentage reduction [$P_{\left(\text{\%}\;reduction\right)}$] in PV power is given by Eq. (2).

(2) $$P_{\left(\text{\%}\;reduction\right)}=\frac{P_{clean}-P_{dirty}}{P_{clean}}\times 100$$

Where $P_{clean}$ = Power of the clean module,

$P_{dirty}$ = Power of the dirty module.

#### 3.1.3 Efficiency

The efficiency of the PV modules is calculated by Eq. (3).

(3) $$\eta_{module}=\frac{P_{max}}{\left(G\times A\right)}$$

Where G = incident radiation flux (W/${\text{m}}^{2}$),

A = Area of the collector (${\text{m}}^{2}$).

#### 3.1.4 Percentage reduction in module's efficiency

The percentage reduction in PV efficiency [$\eta_{\left(\text{\%}\;reduction\right)}$] is given by Eq. (4).

(4) $$\eta_{\left(\text{\%}\;reduction\right)}=\frac{\eta_{clean}-\eta_{dirty}}{\eta_{clean}}\times 100$$

Where $\eta_{clean}$ = Efficiency of the clean module,

$\eta_{dirty}$ = Efficiency of the dirty module.

### 3.2 Dust density measurement setup

**Figure 6:**
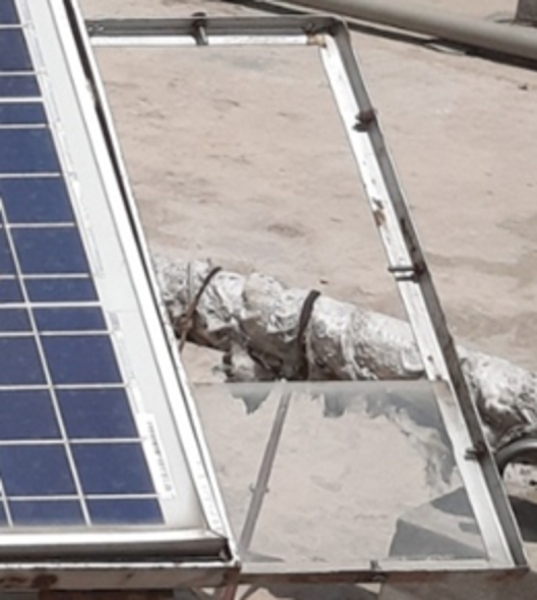
Dust collection setup.

This setup consists of glass sheets mounted on steel supports adjacent to the PV modules as shown in Fig. 6. To measure dust density, dust samples are collected from both locations simultaneously. A digital balance (Radwag AS 220.R2 weighing 0.1 mg of dust with a range of 10 mg to 200 g) is used to weigh glass sheets. To determine the amount of dust on the sheets, accumulated dust is collected from their surfaces and weighed every week. The rate of dust deposition is calculated on weekly basis from the density which is the ratio of dust weight and its surface area. Glass sheet has a known surface area and dust weight is measured by measuring the weight of the glass sheet.

### 3.3 Dust composition and chemical analysis

Chemical and compositional analysis of dust is required to understand the impact that dust has on PV modules. Dust's physical and chemical properties are influenced by various factors including topography, geology and environment; their size and shape influence the dust deposition and PV performance [19]. A detailed analysis of dust's physical properties (size, shape and elemental composition) describes its influence on PV performance. Different sizes and dust types significantly affect solar transmission as each type blocks a unique frequency of solar radiation. Dust analysis can help to design effective prevention techniques. Various aspects of the collected dust are characterized including particle size, surface morphology, mineral composition and chemical composition. Tescan VEGA3 XM scanning electron microscopy (SEM) and Oxford Instruments X-Max 50 Energy-dispersive X-ray spectroscopy (EDS) are used to observe the shape of the sample from PV face and its elemental content. Dust structure is evaluated using a Bruker D8 Superior X-ray diffractometer (XRD) with a Bragg-Bentano θ:2θ configuration in the range of 20^∘^ – 60^∘^. The study has potential limitations because the solar panels are situated in confined place with boundary on all sides due to which factor of wind cannot be assessed. In rainy days, setup is covered with a sheet. Average daily solar radiation is assumed throughout the day. Impact of bird droppings is neglected although it also has drastic impact on PV module.

## 4 Results and discussion

**Figure 7:**
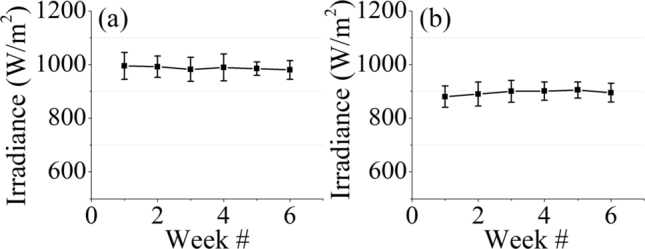
Weekly average solar irradiances at (a) Islamabad and (b) Bahawalpur.

Results have been divided into subcategories as follows: A description of dust measurement and chemical analysis is presented in the first part. The second part reports the effect of dust on the energy yield and operating temperature of the PV module. Solar irradiance for six weeks during this experimental work at both locations is shown in Figs. 7(a) and 7(b). It is measured that PV Module in Bahawalpur captures more solar irradiance with an average value of 986.5 W/m^2^ than the one in Islamabad having average value of 902.46 W/m^2^.

### 4.1 Dust density measurement and chemical analysis

**Figure 8:**
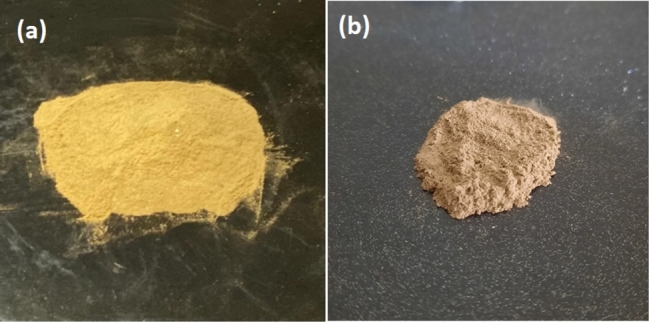
Dust samples for analysis at (a) Islamabad and (b) Bahawalpur.

**Figure 9:**
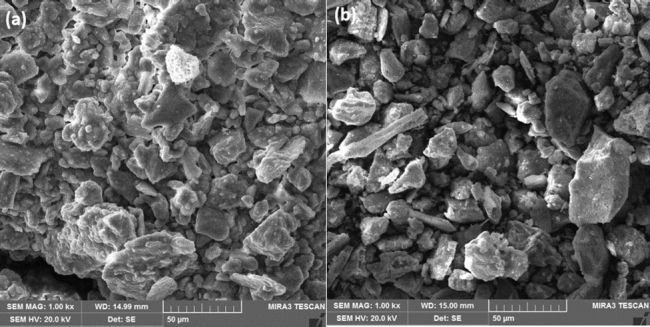
SEM image of dust samples in (a) Islamabad and (b) Bahawalpur.

The accumulated dust on the glass sheets is used to measure dust density which will deduce daily dust accumulation. The experimental results show the dust density after 42 days at a tilt angle of 34.5^∘^ in Islamabad is 6.388 g/m^2^ and in Bahawalpur is 10.254 g/m^2^. Hence, the daily average deposition rates in Islamabad and Bahawalpur are 0.152 g/m^2^ and 0.244 g/m^2^, respectively. It is inferred that the dust deposition in Bahawalpur is much greater than that in Islamabad since Bahawalpur is a desert region and it experiences dust storms frequently. The small amount of collected dust undergoes tests for dust characterization to comprehend the chemical analysis, surface morphology and mineral composition. These dust samples taken for analysis are shown in Figs. 8(a) and 8(b) for Islamabad and Bahawalpur respectively. The surface morphology of dust samples is examined using SEM. It presents that dust particles in Islamabad and Bahawalpur regions have distinct sizes, unsymmetrical shapes and uneven arrangement as shown in Figs. 9(a) and 9(b) respectively.

**Figure 10:**
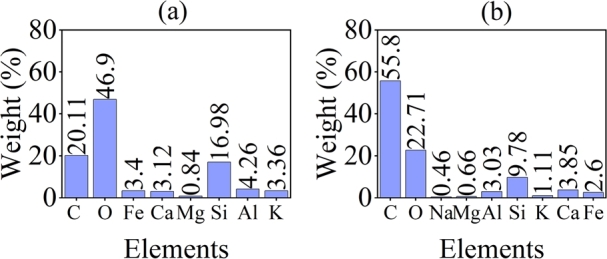
EDS spectrum of dust sample in (a) Islamabad and (b) Bahawalpur.

**Figure 11:**
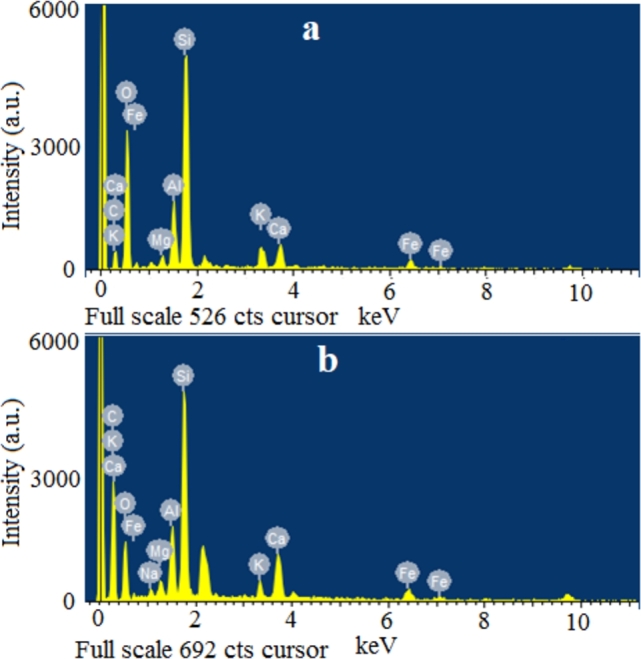
Original EDS plot of dust sample in (a) Islamabad and (b) Bahawalpur.

A dust's elemental composition depends on its location, the pollutants in the region due to nearby activities and the garbage type from the habitats of the locality. To determine the elemental composition, EDS is performed on the dust samples. The results in Fig. 10(a) show that, in the dust sample collected from Islamabad, carbon dominates with 55.8% composition followed by oxygen, silicon and calcium with 22.71%, 9.78% and 3.85% composition respectively. Aluminium, iron, potassium, magnesium and sodium are also found in significantly fewer quantities. Whereas, in the dust sample collected from Bahawalpur shown in Fig. 10(b), oxygen leads to a 46.9% composition followed by carbon, silicon and aluminium with compositions of 20.11%, 16.98% and 4.26% respectively. Original EDS plot is shown in Figs. 11(a) and 11(b). Small contents of calcium, iron, magnesium and potassium are detected.

**Figure 12:**
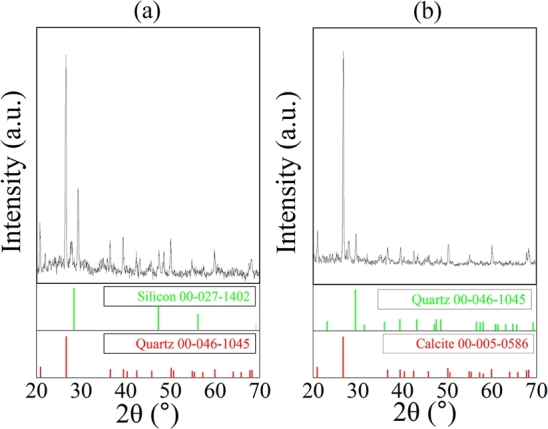
XRD of dust sample at (a) Islamabad and (b) Bahawalpur.

The XRD results obtained by bouncing off of x-rays from the sample into the x-ray detector are represented in Figs. 11(a) and 11(b). It shows that the main components of dust samples in Islamabad are silicon and quartz and the dust sample from Bahawalpur mainly consists of quartz and calcite. Peaks were identified and matched using X'PERT HIGHSCORE software. In Fig. 12(a), green peaks and red peaks represent the peaks from reference library of silicon and quartz, respectively. The highest peak belongs to quartz. Fig. 12(b) shows that highest peak belongs to Calcite. Reference library peaks of quartz and calcite are shown in green and red respectively. Islamabad is an urban area with more traffic and industries which is why the carbon ratio is much higher in the dust sample collected. Carbon dioxide is a main waste product of automobiles and industries. Deposition of carbon dust can cause more power loss than partial shading [32]. Carbon element is not much dominating in dust sample taken from Bahawalpur.

### 4.2 Effect of dust on the energy yield and operating temperature

**Figure 13:**
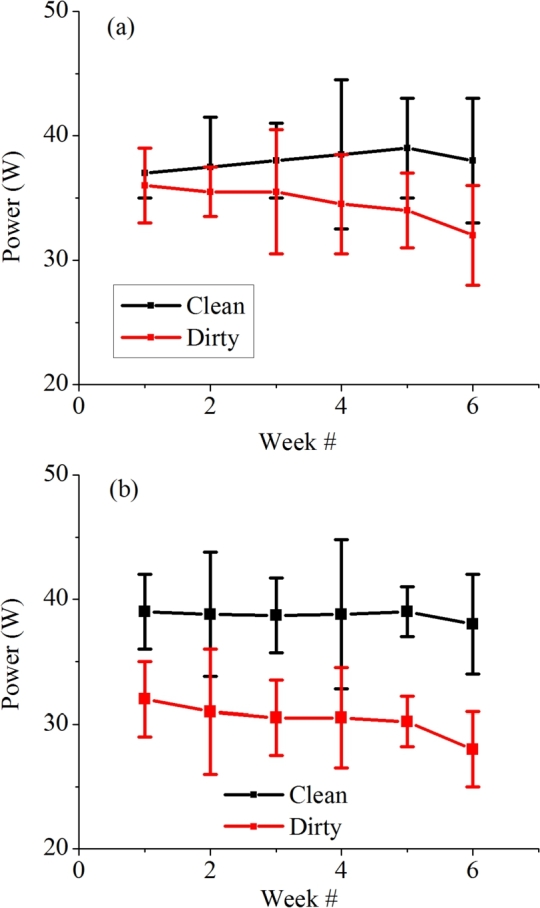
Average weekly output power of module in (a) Islamabad and (b) Bahawalpur.

The performance of a PV module is mainly deteriorated by dust accumulating on its surface. The performance of the module is monitored only in three clear days of a week at the same time to ensure that the solar irradiance is same and parameters are not affected by it. The parameters of the PV module used to analyze the performance at both locations include (i) dust density, (ii) output power, (iii) percentage reduction in power and (iv) temperature. It is observed that dust affects the output power of PV modules by comparing the output powers of clean and dirty modules in both locations. It can be observed from the graphs in Figs. 13(a) and 13(b) that the output power of the dirty module in contrast to the clean module decreases over time. However, the difference in output power of modules placed in Bahawalpur is significantly higher.

**Figure 14:**
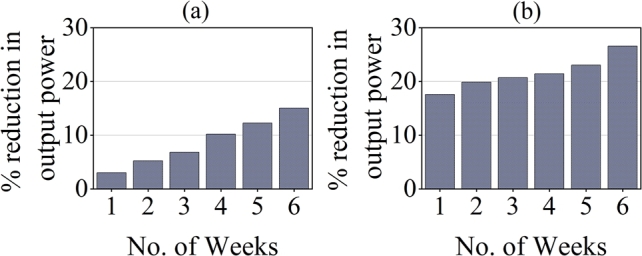
Average percentage reduction of output power of dirty module in contrast to clean module at (a) Islamabad and (b) Bahawalpur.

Similarly, Figs. 14(a) and 14(b) demonstrate that the percentage reduction of the output power of the dirty module in contrast to the clean module is much higher at Bahawalpur. This is because the dust accumulation rate is higher in this region. Chen et al. reported that the output power of PV modules decreases by 7.4% for 0.644 g/m^2^ dust density in a week [33]. Mustapha et al. reported 8.41% loss in output power after dust deposition on PV panel surface in Saharan environment [34]. Dust deposition also causes partial shading of the PV module due to which hot-spot heating may occur. Hot-spot heating areas are areas with high temperature that reduce the efficiency of PV module. They are caused when there is low current in any cell due to shading while other cells perform normally [35]. The excess electricity leads to formation of hotspots. This causes aging of PV module, cracking or melting of cells and can even cause fire [36]. However the span or our study was short to detect any such problem and latest PV technologies are working to mitigate this issue.

**Figure 15:**
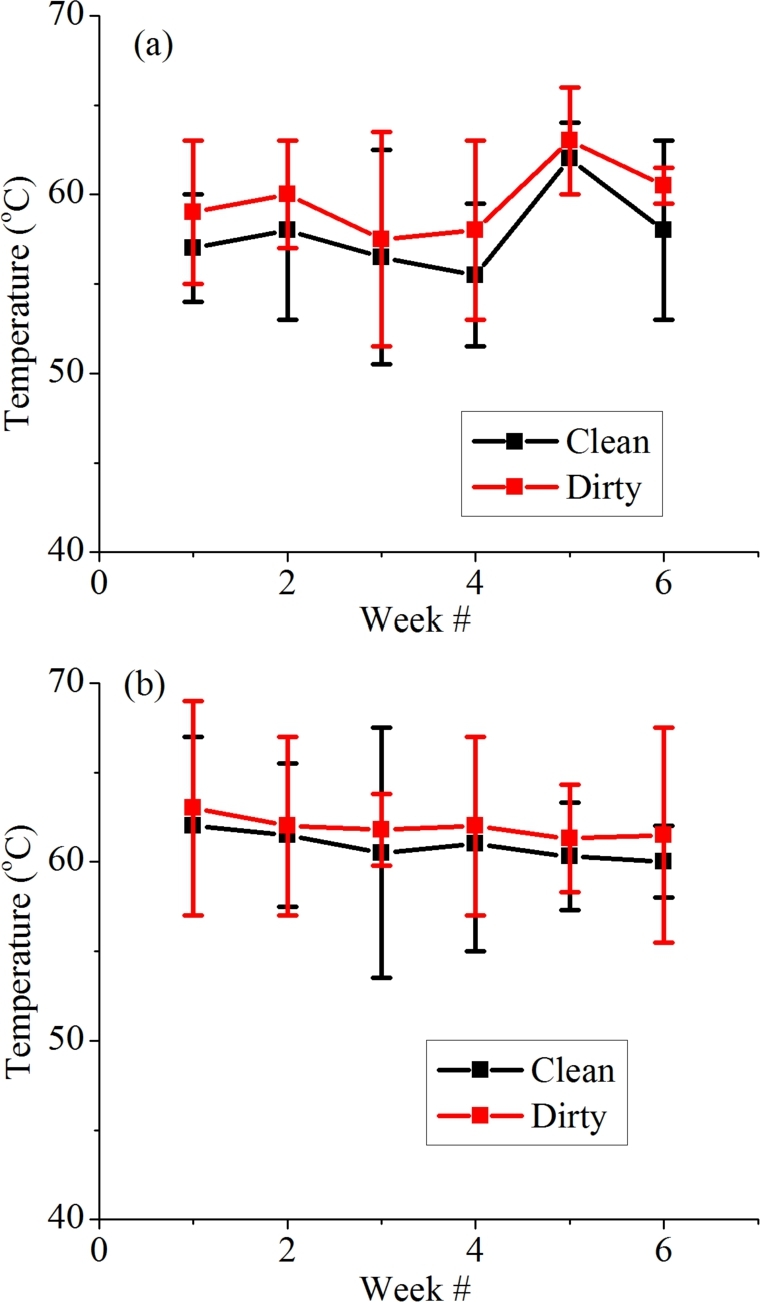
Average weekly temperature of dirty and clean PV module (a) Islamabad and (b) Bahawalpur.

A PV module's output also depends on the module's temperature. A PV module is rated at 25 ^∘^C, but when operating in the field, they generally operate at higher temperatures. Fig. 15(a) represents the temperature of clean and dirty modules in Islamabad region and that in Bahawalpur region is shown in Fig. 15(b) and both plots show that the dust deposition leads to a rise in temperature of PV modules in both cities. PV modules in Bahawalpur show a more significant temperature rise as compared to that in Islamabad. This may be due to the high rate of dust accumulation or because the temperature in Bahawalpur is generally higher.

**Figure 16:**
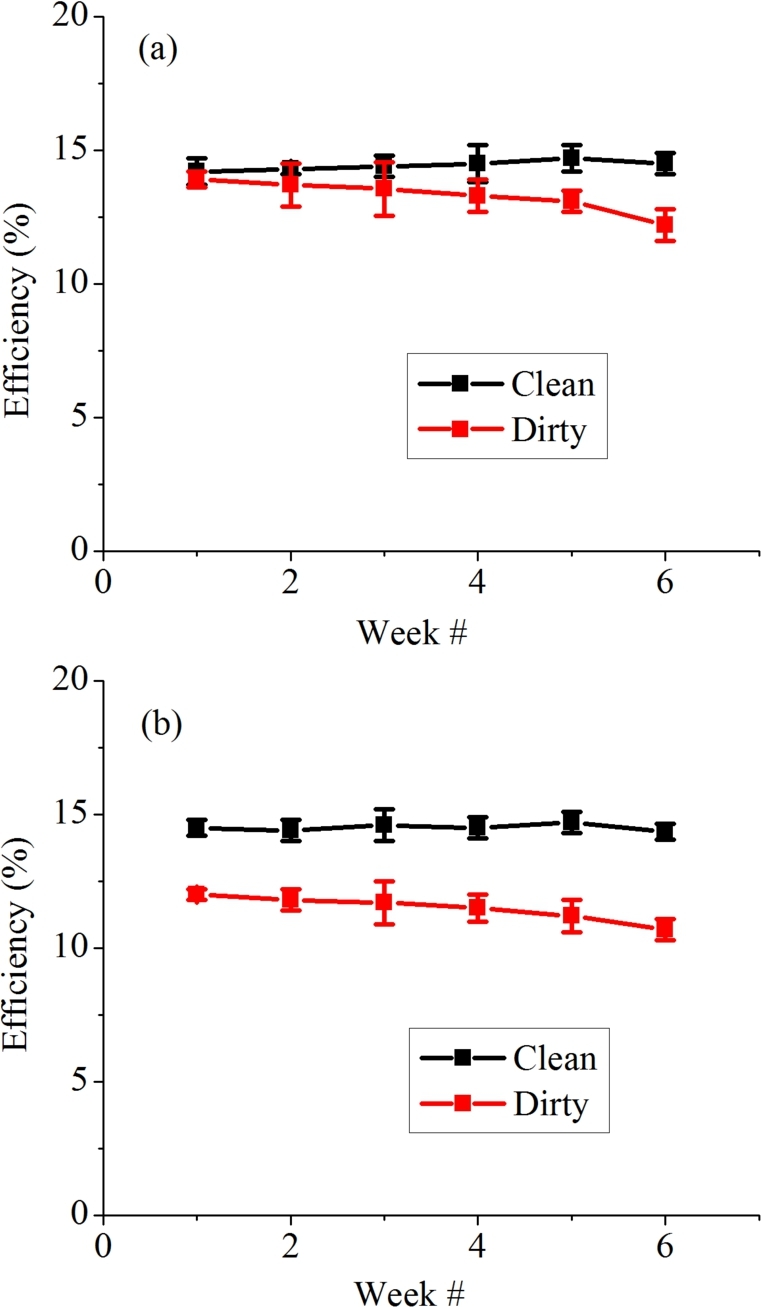
Average weekly efficiency of dirty and clean PV module at (a) Islamabad and (b) Bahawalpur.

A spike in temperature of PV module located in Islamabad on week five is due to higher solar irradiance in that week. A PV module's electrical efficiency is decreased when it is overheated [37]. It can be validated from graphs shown in Figs. 16(a) and 16(b) that the efficiency of PV modules in Bahawalpur reduced drastically as compared to that in Islamabad region. Rahman et al. reported reduction of 3.13% in module's efficiency as temperature of module reaches up to 56 ^∘^C [38]. Benghanem et al. reported that with decrease in every degree Celsius there is increase of 0.5% in solar cell efficiency [39]. According to the results and literature it can be concluded that the temperature of module has a huge impact on output of the module. The effect of dust accumulation on PV modules has been studied very briefly by them. Efficiency of solar panels is also reduced by dust deposition. Salari et al. reported similar results and according to them, as dust density increases from 0 g/m^2^ to 8 g/m^2^, efficiency of PV module decreases by 26.36% [40]. All the obtained results verify a significant drop in the output efficiency of PV modules located in Bahawalpur. The dust characterization shows presence of small particles in the sample, dust density results shows a high amount of deposition in same span. This leads to an inefficient power generation and rise in temperature of PV module.

## 5 Conclusion

In this paper, a comprehensive analysis is carried out to draw a comparison of the impact of dust accumulation on the performance of PV module associated with different climatic conditions, the composition of dust and the amount of dust.

• The results from the experimental setup indicate that, after six weeks of uncleaned PV modules, the reduction in output power was 15.08% in Islamabad, whereas 25.42% in Bahawalpur, *i.e.*, around 1.5 times higher than that in Islamabad.
• Islamabad, an industrial zone with average temperature and high rainfall, received 6.388 g/m^2^ dust on the solar panel at a daily average deposition rate of 0.152 g/m^2^. On the other hand, Bahawalpur, a dry and primarily dusty region, received 10.254 g/m^2^ dust on the solar panel at a daily average deposition rate of 0.244 g/m^2^.
• The SEM analysis and power values obtained show that smaller particles block more sunlight and reduce PV module's efficiency.
• Further dust characterization results indicate that both dust samples have a high percentage of oxygen, carbon and silicon.
• The temperature of the module mainly depends on ambient temperature. It is also concluded that the temperature of the dirty module is higher than that of the clean module. This is due to the dust-temperature phenomenon, *i.e.*, the dust layer traps the sunlight and increases the module's temperature resulting in decreased efficiency.

The results indicate that being a sunny region, Bahawalpur has excellent potential for solar power as the modules installed there show higher efficiency. However, the high dust deposition rate in Bahawalpur, the percentage reduction in output power and the module's temperature are much more significant. Hence, the Bahawalpur dust is more lethal for PV modules. As a result, the high dust deposition rate in Bahawalpur, the percentage reduction in output power, and the module's temperature are much more significant. Therefore, a proper cleaning schedule and system are required for efficient solar power production.

## CRediT authorship contribution statement

**Mahnoor Rashid:** Writing – original draft, Methodology, Data curation. **Muhammad Yousif:** Supervision, Resources, Project administration, Investigation. **Zeeshan Rashid:** Writing – review & editing, Formal analysis. **Aoun Muhammad:** Conceptualization. **Mishal Altaf:** Visualization, Software. **Adil Mustafa:** Validation, Supervision, Funding acquisition.

## Declaration of Competing Interest

The authors declare that they have no known competing financial interests or personal relationships that could have appeared to influence the work reported in this paper.

## Data Availability

Data included in article/supp. material/referenced in article.
